# The effect of a topical curcumin formulation (VAS-101) on knee pain in adults with knee osteoarthritis: a randomised, double-blind, placebo-controlled study

**DOI:** 10.3389/fpain.2026.1789088

**Published:** 2026-04-01

**Authors:** Adrian L. Lopresti, Benny Antony, Stephen J. Smith

**Affiliations:** 1 Clinical Research Australia, Perth, WA, Australia; 2 College of Science, Health, Engineering and Education, Murdoch University, Perth, WA, Australia; 3 Menzies Institute for Medical Research, University of Tasmania, Hobart, TAS, Australia; 4 Faculty of Medicine and Health, Kolling Institute, The University of Sydney, St Leonards, NSW, Australia

**Keywords:** clinical trial, curcumin, knee osteoarthritis, pain, topical, turmeric

## Abstract

**Background:**

The oral delivery of curcumin has been shown in several studies to have beneficial pain-relieving effects for the treatment of knee osteoarthritis. However, there has been limited investigation into its efficacy and tolerability when delivered topically. The purpose of this two-arm, 28-day, parallel-group, randomised, double-blind, placebo-controlled trial was to determine the effects of a topical curcumin gel (VAS-101) on knee pain and symptoms in adults with knee osteoarthritis.

**Methods:**

Sixty adults aged 45–75 with knee osteoarthritis applied a curcumin or placebo gel to their knee, every second day for 28 days. Outcome measures comprised the Knee Injury and Osteoarthritis Outcome Score (KOOS), daily pain ratings, and several performance-based tests. Rescue oral medication intake was also monitored over time.

**Results:**

Compared to the placebo, VAS-101 was associated with greater improvements in the KOOS pain score (primary outcome measure) (β: 5.12; 95% CI: 0.47, 9.77; d = 0.62, *p* = 0.041), and mean daily pain ratings (F_3, 225_ = 4.42; d = 0.55, *p* = 0.005). In the VAS-101 group, 39.3% of participants reported feeling either much or very much improved, compared with 13.3% in the placebo group (*p* = 0.019). Moreover, 32.1% of participants in the VAS-101 group achieved a Minimal Clinically Important Difference, compared to 13.3% in the placebo group, although this group difference was not statistically significant (*p* = 0.086). There were no group differences in changes in other KOOS subscale scores or the performance-based tests. VAS-101 was well-tolerated, with no significant adverse reactions reported. However, skin staining was observed as expected with topical curcumin, which resolved 2–3 days after application ceased.

**Conclusions:**

Conservative dosing of a topically applied curcumin-containing gel (VAS-101), administered every two days for 28 days, is associated with moderate reductions in knee pain in adults with knee osteoarthritis. Further investigations utilising larger sample sizes, longer treatment durations, and alternative treatment regimens will be important to identify how these factors affect treatment adherence, tolerance, and efficacy.

## 1 Introduction

Osteoarthritis (OA) is the most common musculoskeletal disease globally, which leads to significant disability arising from reduced joint mobility, increased functional burden, and reduced quality of life (1). Knee OA accounts for approximately 85% of OA cases worldwide (2). It is a multifactorial disease characterised by several pathological changes, including cartilage degradation, osteophyte formation, remodelling of osteo-cartilaginous units, and joint inflammation (3). Common pharmacological treatment strategies for knee OA include oral analgesics such as acetaminophen, oral non-steroidal anti-inflammatory drugs (NSAIDs), topical NSAIDs, and intra-articular corticosteroid injections. Although these treatments have demonstrated modest efficacy, there remains a significant portion of individuals who fail to experience significant relief from such treatments, experience adverse effects, or have comorbid medical conditions that make some of these treatments contraindicated (3, 4).

Curcumin, a polyphenolic compound derived from turmeric rhizomes, is an anti-inflammatory compound often used to treat pain-related conditions, including OA. In a recent meta-analysis, it was concluded that, based on 23 studies and 2,175 patients with knee OA, compared with placebo, oral curcumin reduced self-reported pain as measured by the visual analogue scale and the Western Ontario and McMaster Universities Arthritis Index. Curcumin was also associated with a reduction in the use of pain-relieving medication for the treatment of knee pain (i.e., NSAIDs-sparing rescue medication) (5). Similar conclusions were also reported in a meta-analysis examining the effects of high and low-dose curcumin on knee OA (6).

However, a commonly reported problem associated with oral curcumin ingestion is its poor bioavailability, which is believed to limit its therapeutic efficacy (7). This has resulted in various methods to increase its solubility and absorption, including creating micro- and nano-formulations, combining it with turmeric oil, piperine, or delivering it as a turmeric oleoresin (8). While such methods have improved bioavailability, further research is required to determine if this enhanced bioavailability is associated with greater clinical efficacy. Moreover, large oral doses of curcumin have been reported to cause gastrointestinal upset and/or liver damage (9, 10). Conversely, there has been little investigation into the pain-relieving efficacy of curcumin when delivered topically or through transdermal applications. Using an OA mouse model, the topical administration of curcumin suppressed the expression of several pro-inflammatory mediators and upregulated the chondroprotective transcriptional regulator CITED2 in primary cultured chondrocytes. Moreover, in contrast to oral curcumin, its topical delivery was associated with a reduction in OA-related pain as indicated by reduced tactile hypersensitivity and improved locomotor behaviour (11).

VAS-101 (also known as Vasceptor®) is a topical curcumin gel formulation that utilises a patented topical/ transdermal delivery platform designed to avoid first-pass liver metabolism and to increase the bioavailability of curcumin. A preclinical study demonstrated that the transdermal delivery of VAS-101 was absorbed and entered the circulation, resulting in measurable drug levels (12). Using a sickle cell mouse model, curcumin was detected in the blood for up to 6 h after transdermal delivery and was associated with reduced cold pain sensitivity after 1 day, an effect that persisted for the 21-day testing period. Based on this animal proof-of-concept investigation, this study aimed to assess the clinical efficacy and safety of VAS-101 administration for 4 weeks in adults with knee OA. It was hypothesised that VAS-101 would be associated with reduced knee OA symptoms, including knee pain, and improved functional mobility.

## 2 Materials and methods

### 2.1 Study design and procedures

Ethics approval was obtained from the National Institute of Integrative Medicine Human Research Ethics Committee, and informed consent was obtained from participants prior to the commencement of the study. This study was registered prospectively on the 16th January 2025 with the Australian and New Zealand Clinical Trials Registry (ACTRN12625000037404).

**Figure 1:**
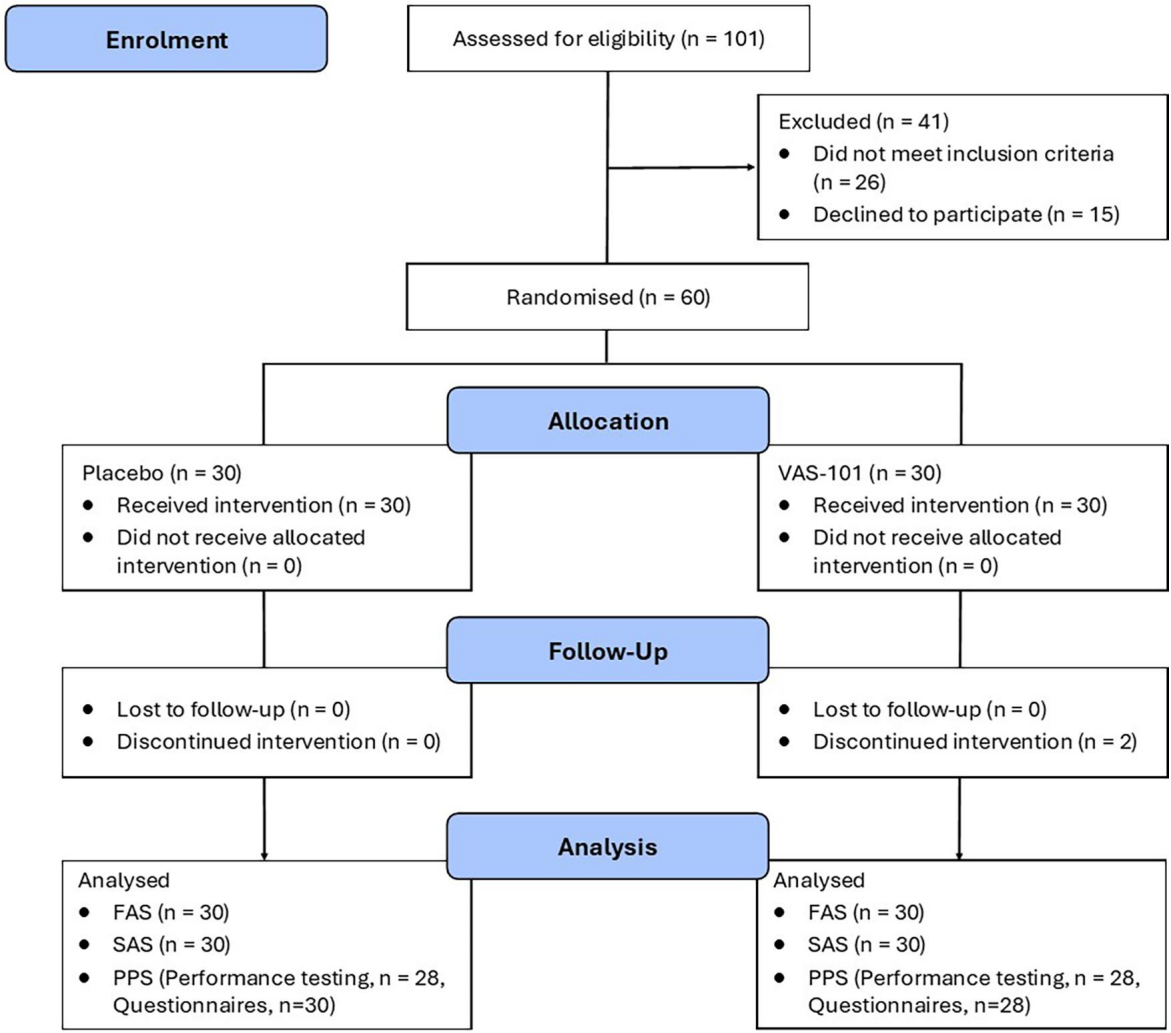
Systematic illustration of study design.

This was a 4-week, two-arm, parallel-group, randomised, double-blind, placebo-controlled trial (Figure 1). Participants completed an online screening questionnaire and, if potentially eligible, underwent a telephone interview with a researcher to obtain further details regarding the eligibility criteria. If willing and eligible to participate, participants underwent an x-ray of their target knee, during which a Kellgren-Lawrence (KL) grade (ranging from 0 to 4) was assigned. Participants also provided written confirmation of a diagnosis of their knee OA, completed by a medical practitioner. Visit 1 (day 0) was scheduled approximately 7–14 days after the telephone interview. The target knee was determined as the most painful knee. However, if both knees had similar pain, the most dominant leg was established as the target knee and had to meet all the eligibility criteria. During visit 1, the following tasks were completed: (1) measurement of weight, height, resting pulse, and blood pressure, (2) completion of a performance-based assessment comprising the 30-second Chair Stand Test, 40-metre Fast-Paced Walk Test, Timed Up and Go Test, and Six-minute Walk Test (as per procedures published by the Osteoarthritis Research Society International (13), (3) participant completion of self-report questionnaires, (4) instruction and demonstration on how to apply the gel to the target knee, and (5) 4-week provision of the investigational product (placebo or curcumin). Participants were requested to not engage in any exercise on the day of each visit and to refrain from participating in any vigorous exercise during the study period. Similar procedures were undertaken on visit 2 (day 14) and visit 3 (day 28). Moreover, participants were required to complete self-report questionnaires (online) on days 7 and 21, and every evening at approximately 8 pm, they were required to provide a daily rating of their overall knee pain while walking and list any oral pain-relieving medication taken for their knee pain.

### 2.2 Participants

#### 2.2.1 Inclusion criteria

Inclusion criteria for the study comprised the following: healthy male and female adult; aged between 45 and 75 years; had knee OA defined according to the National Institute for Health and Care Excellence clinical criteria (age 45 years or older, has activity-related knee joint pain, and has no morning knee stiffness or stiffness of 30 min or less); a body mass index (BMI) between 18 and 32 kg/m^2^; able to provide evidence of a previous diagnosis of knee OA by a medical practitioner; at the time of screening, reported an average knee pain while walking over the last week of 4–7 on a 10-point numeric rating scale (1 = no pain to 10 = worst pain possible); reported experiencing knee pain on more than 50% of days over the previous month; and had no plan to commence new treatments over the study period.

#### 2.2.2 Exclusion criteria

The exclusion criteria comprised the following: recently diagnosed or unmanaged medical conditions, including but not limited to, cardiovascular disease, diabetes, gastrointestinal disease, endocrine disease, neurological disease, cancer/malignancy, or in the opinion of the investigator, had other conditions that would jeopardise safety or impact validity of results; had arthritis of the hip or chronic back pain that significantly affected daily function; had an intra-articular treatment/injections with a corticosteroid or hyaluronic acid in the target knee within 6 months before screening; had surgery on the target knee within 6 months before screening; had a planned surgery during the study period; had a clinically significant infection, injury, or illness within 28 days before screening; planned to engage in heavy exercise (e.g., marathon run, heavy leg squats) during the study; had a known allergic reaction to curcumin-containing products; had an injury in the area of the target knee within 3 months before screening; was taking curcumin-containing products; commenced or changed pharmaceutical medication, herbal or vitamin supplements within 4 weeks before screening; consumed more than 14 standard serves of alcohol per week; had a 12-month history of regular illicit drug use; or was pregnant, breastfeeding, or intended to become pregnant in the next 2 months.

### 2.3 Randomisation and blinding

Through social media advertisements and emails to an in-house database, volunteer recruitment occurred from February to June 2025. Eligible participants were randomly allocated to either a VAS-101 or placebo condition in a 1:1 ratio using a randomisation calculator. The randomisation structure consisted of 6 blocks, with 10 participants per block, and was completed by a study sponsor not involved in volunteer recruitment or visit assessments. Identification numbers were allocated based on the order of enrolment. The investigational product was provided in a semi-opaque, orange dispenser. Details of group allocations were held by the study sponsor, with researchers and the statistician remaining blinded to group allocation until all study outcomes were obtained and a blind review was undertaken.

### 2.4 Interventions

The intervention consisted of either a curcumin-containing gel (VAS-101) or a placebo gel. The placebo and active gel were yellow and had a similar consistency. The curcumin extract used in VAS-101 was Curcugen® (manufactured by Dolcas-Biotech LLC, Bikaner, India). Curcugen® contains 50% curcuminoids (curcumin, desmethoxycurcumin, and bis-desmethoxycurcumin), with 8 mg of Curcugen® delivered per dose (0.1 mL). The placebo gel contained USP/NF-grade yellow dye, and the excipients in both gels comprised polyethylene glycol 400 and myristic acid. Participants were required to apply 0.1 mL of the gel (equivalent to two clicks on the dispenser) to the side of the target kneecap (the peripatellar fat pads) and directly on the target kneecap every other day. After application, the gel was rubbed into the skin surrounding the kneecap and directly onto the kneecap using a toothbrush that was supplied to each participant. Brushing into the skin lasted at least 1 min. A preclinical study demonstrated that the transdermal delivery of 0.1 mL of VAS-101 in mice was absorbed and entered the circulation, resulting in measurable drug levels in the blood for up to 6 h (12). The dose of 0.1 mL, every second day, was selected for this study based on results from this preclinical trial and consumer feedback from those who have applied the gel. It also reflects a practical volume that can be easily applied to the knee without causing excessive skin saturation or dripping.

After application, a loose-fitting knee sleeve was applied to reduce the chances of staining and participants were instructed not to shower or bathe for 12 h post-application. In a preclinical study, detectable curcumin levels in blood were observed up to 6 h after application, so a 12-hour application was recommended to maximise curcumin absorption through the skin. Participants returned their gel dispenser at visits 2 and 3. Treatment compliance was measured by the completion of a daily questionnaire, in which participants indicated whether they had applied the gel. To evaluate treatment blinding, participants were asked to predict their group allocation (placebo, curcumin, or unsure) at visit 3.

### 2.5 Outcome measures

#### 2.5.1 Knee injury and osteoarthritis outcome score (KOOS) pain score

The KOOS is a validated self-report questionnaire designed to assess short and long-term patient-relevant outcomes following knee injury/pain (14, 15). It comprises 42 items and calculates sub-scale scores for pain (9 questions), symptoms (7 questions), activities of daily living (17 questions), sports and recreation function (5 questions), and quality of life (4 questions). The change in the KOOS pain score from baseline to day 28 was the primary outcome measure. The KOOS was completed on days 0, 7, 14, 21, and 28.

#### 2.5.2 Daily numeric pain rating scale (NPRS)

Participants provided an overall rating of their daily knee pain while walking (1 = no pain to 10 = worst pain possible) every evening from days 0 to 28.

#### 2.5.3 Performance-based tests

Tasks recommended by the Osteoarthritis Research Society International (OARSI) to assess physical function in people diagnosed with hip or knee osteoarthritis (13) were completed on days 0, 14, and 28. The following tasks were completed by participants: the 30-second Chair Stand Test (number of repetitions), the 40-metre (4 × 10 m) Fast-Paced Walk Test (time to complete in seconds), the Timed Up and Go Test (time to complete in seconds), and the Six-minute Walk Test (distance completed in metres).

#### 2.5.4 Patient global impression of change (PGIC)

The PGIC reflects a person's belief about the efficacy of treatment and is widely used in chronic pain clinical trials (16). On days 14 and 28, participants estimated the difference between their current and previous health state using a Likert scale from (1) very much improved to (7) very much worse.

#### 2.5.5 Rescue medication intake

Participants recorded their daily intake of oral pain-relieving medication used to reduce pain in the index knee (rescue medication) every evening. The type and number of pills taken were recorded.

#### 2.5.6 Safety and expectancy measures

The tolerability of the gel application was evaluated through weekly questionnaires enquiring about adverse events and interviews at visits 2 and 3. The Patient Global Assessment of Tolerability to Therapy (PGATT) was also completed at visits 2 and 3, where participants indicated how well they tolerated the gel from ‘Worst—I experienced severe discomfort & was unable to tolerate the treatment’ to Excellent—I experienced no discomfort or adverse effects. Resting pulse and seated blood pressure were also measured on days 0, 14, and 28. As expectancies can influence treatment outcomes in placebo-controlled (17), participants completed the Clinical Trials Treatment Expectancies Scale (CTTES) at visit 1. The CTTES, a 6-item questionnaire, has been used in previous trials to measure and control treatment expectancies (18).

### 2.6 Sample size calculations

A total of 60 participants commenced this two-treatment, parallel-design study, assuming a 10% dropout rate. The probability was 80 per cent that the study would detect a treatment difference at a two-sided 0.05 significance level, if the true difference between treatments was 15.000 units (the minimal clinically important difference on a 100-point scale). This was based on the assumption that the standard deviation of the response variable was 19 (19).

### 2.7 Statistical analysis

Outcome analyses were conducted on the full analysis set (FAS) and per protocol set (PPS), with all participants retained in their originally assigned groups. The FAS is the subset of participants who were randomised and administered at least one dose of the trial product and who had available efficacy data. The PPS is the subset of participants who were randomised, administered at least one dose of the trial product, had available efficacy data, and had no major protocol deviations.

Generalised Linear Mixed Models (GLMM) assessed differences between intervention groups for treatment outcomes comprising the KOOS, NPRS, and performance testing. No manual imputation was used for missing data, as the GLMM handles them via maximum likelihood estimation. To examine between-group differences, changes in scores from day 0 to day 28 were calculated, and GLMM were used to examine between-group differences in these scores. Corresponding baseline scores were included as covariates. Moreover, as age (20–22), sex (23, 24), and BMI (25, 26) can significantly affect the severity, treatment efficacy and pathophysiology associated with OA, these variables were included as covariates. Treatment expectations can also significantly affect the placebo response in pain treatments, so the CTTES positive expectancies score was included as a covariate (27, 28). To examine changes over time, a GLMM was used, incorporating all time points. This comprised days 0, 7, 14, 21 and 28 for KOOS scores, and Days 0, 14, and 28 for scores obtained on performance testing. Target distributions, relationship (link), and covariance structures were selected to create the best-fitting model using Bayesian modelling. Random intercepts were utilised in each model, and covariates age, sex, BMI, and CTTES positive expectancy score were included (fixed effects). Means NPRS were calculated for each week (weeks 1, 2, 3, 4), and between-group differences (time × group interaction) and within-group changes (time effects) were analysed using GLMM. Estimated marginal means are based on the original target scale, and simple contrasts were used to examine time and group contrasts. For each outcome measure, simple contrasts were adjusted for multiple comparisons using the Sequential Bonferroni procedure. As a *post-hoc* analysis, using criteria established by Jacquet et al. (29), a Minimal Clinically Important Difference (MCID) of 15.4 was determined. Therefore, participants experiencing a change in the KOOS pain score of more than 15.4 from day 0 to day 28 were categorised as experiencing an MCID. A chi-square test was conducted to examine group differences in the number of participants who achieved an MCID. As data were not normalised, group differences in rescue medication use were analysed using an Independent-Samples Mann–Whitney U test. This comprised an analysis of the percentage of rescue medication-free days (i.e., the number of days without taking a medication over 28 days) and the total number of rescue medication pills taken (across all types) over the 28-day period. Group differences in PGIC and PGATT ratings on days 14 and 28 were analysed using an independent samples Mann–Whitney U Test. For safety outcomes, group differences in changes in weight, blood pressure and pulse were analysed using a repeated-measures ANOVA. All data were analysed using SPSS (version 28; IBM, Armonk, New York, USA). As there was only one *a priori* primary endpoint, the critical *p*-value was set at *p* ≤ 0.05 (two-sided) for all analyses. However, a hierarchical order of secondary efficacy endpoints is detailed in Supplementary Table S1, in which statistical significance was no longer reported after the first endpoint failed to reach statistical significance (*p* > 0.05).

## 3 Results

### 3.1 Study population

As detailed in Figures 1, 101 people were screened, and 60 were randomly assigned to a treatment condition. The reasons for ineligibility were failure to meet eligibility criteria (*n* = 26) and withdrawal of consent to participate after the telephone interview (*n* = 15). Baseline sociodemographic and clinical characteristics, as well as mean scores for the assessments at Visit 1, are presented in Table 1. There were no differences in treatment expectations at baseline as measured by the CTTES positive (*p* = 0.598) or negative (*p* = 1.000) scores.

**Table 1:** Baseline sociodemographic and clinical characteristics.

Sociodemographic and clinical characteristics		Placebo (*n* = 30)	VAS-101 (*n* = 30)
Age (yrs)	Mean	62.46	61.10
	SD	6.42	7.83
Sex (n)	Male	10	13
	Female	20	17
Height (m)	Mean	1.71	1.70
	SD	0.07	0.10
Weight (kg)	Mean	78.88	79.00
	SD	12.59	13.82
BMI (kg/m2)	Mean	27.01	27.09
	SD	3.20	3.09
Systolic blood pressure (mmHg)	Mean	129.03	131.93
	SD	17.12	14.72
Diastolic blood pressure (mmHg)	Mean	78.33	80.23
	SD	9.54	7.22
Pulse (bpm)	Mean	67.93	70.73
	SD	10.53	11.80
Marital Status (n)	Single	9	7
	Married/ defacto	21	23
Education (n)	Secondary	14	17
	Tertiary	8	8
	Post-graduate	8	5
Kellgren-Lawrence (KL) grade	Grade 0	0	0
	Grade 1	3	5
	Grade 2	11	9
	Grade 3	13	16
	Grade 4	3	0
Occupation (n)	Retired	11	11
	Technicians and associated trades	7	3
	Professional	3	5
	Services and sales worker	3	4
	Clerical support worker	3	2
	Craft and related trades worker	2	2
	Manager	1	2
	Unemployed	0	1
Knee pain rating at screening (0–10)	Mean	5.37	5.40
	SD	1.27	1.19
Target knee	Left knee	14	15
	Right knee	16	15
KOOS—Symptoms Score	Mean	61.57	61.30
	SD	12.94	15.25
KOOS—Pain Score	Mean	60.67	60.47
	SD	15.58	12.57
KOOS—Daily Living Score	Mean	67.10	70.87
	SD	16.04	14.29
KOOS—Sports & Recreation Score	Mean	36.33	39.67
	SD	19.52	19.30
KOOS—Quality of Life Score	Mean	39.30	45.53
	SD	17.37	13.89
CTTES positive expectancies	Mean	9.10	9.37
	SD	2.12	1.75
CTTES negative expectancies	Mean	3.33	3.33
	SD	1.12	0.76
Chair-Stand Test (Repetitions)	Mean	14.57	13.50
	SD	5.42	4.31
Paced walk test (secs)	Mean	27.43	26.94
	SD	4.80	4.42
Time-up and go test (secs)	Mean	7.41	7.54
	SD	1.37	1.69
Six-minute walk test (metres)	Mean	499.50	521.77
	SD	69.00	60.79

### 3.2 Outcome measures

#### 3.2.1 Pain scores

**Figure 2:**
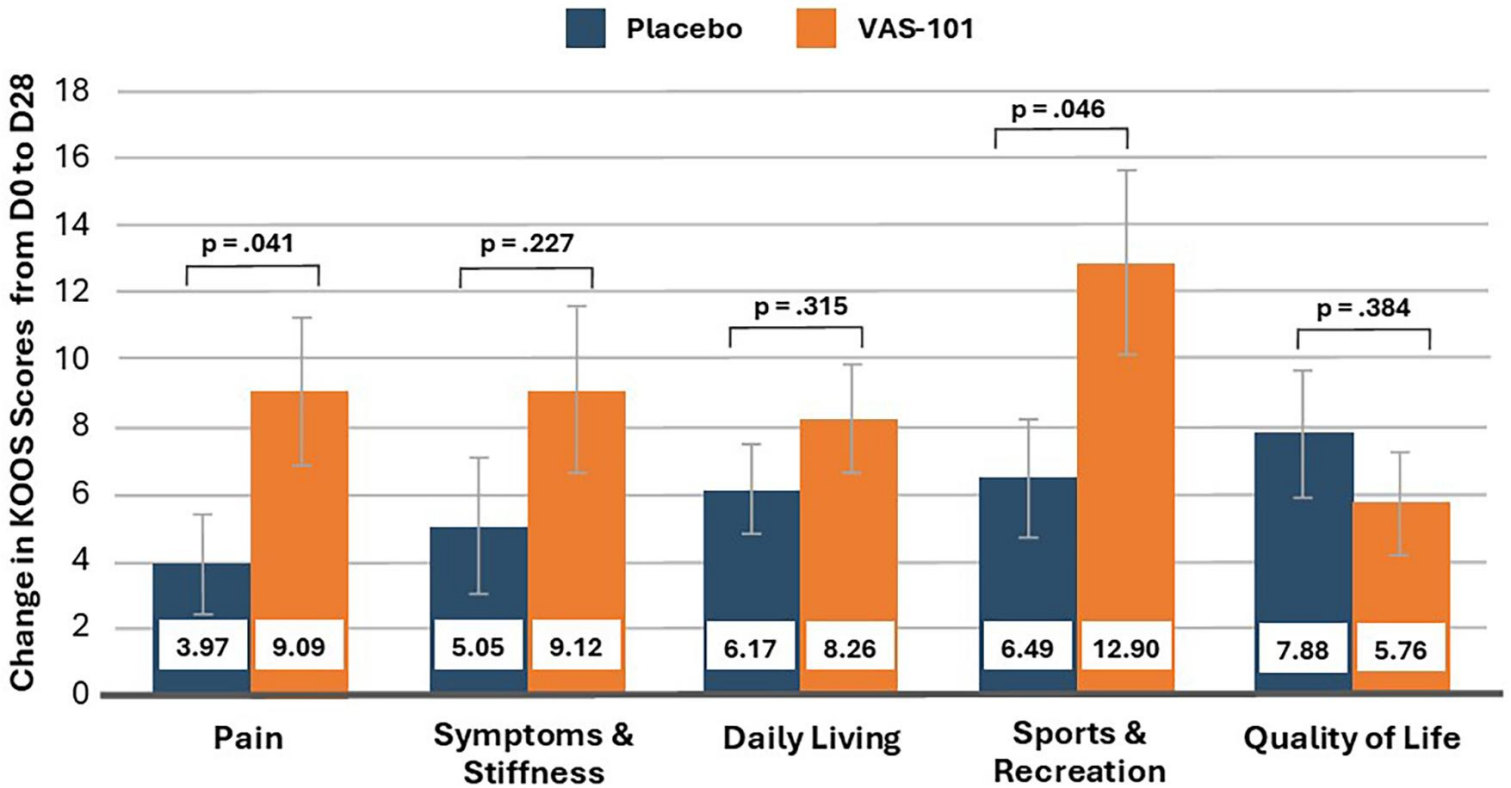
Change in KOOS scores from Day 0 to Day 28 (vertical bars represent standard errors) (estimated marginal means).

As demonstrated in Table 2 and Figure 2, there was a statistically significant group difference in the change in the KOOS pain score (primary outcome measure) from baseline to day 28 (β: 5.12; 95% CI: 0.47, 9.77; d = 0.62, *p* = 0.041). In the VAS-101 group, the pain score increased (indicating a reduction in pain) by a mean of 9.09 points (95% CI: 5.64, 14.64), and in the placebo group, it increased by 3.97 points (95% CI: 1.87, 8.44). After controlling for baseline scores (mean: 60.98), the KOOS pain score increased from baseline to day 28 by 14.9% in the VAS-101 group and by 6.5% in the placebo group. Based on the PPS (Supplementary Table S2), similar group differences in changes in pain scores were identified.

**Table 2:** Change in KOOS scores (estimated marginal means) (FAS).

KOOS Scores		Placebo (*n* = 30)							VAS-101 (*n* = 30)							*P*-value^b^	*P*-value^c^	Cohen's D
		Day 0	Day 7	Day 14	Day 21	Day 28	Change D0 to 28	*P*-value^a^	Day 0	Day 7	Day 14	Day 21	Day 28	Change D0 to 28	*P*-value^a^			
KOOS pain score	Mean	61.06	65.30	64.91	67.32	66.46	3.97	0.053	60.40	67.21	67.29	70.34	71.08	9.09	<.001	0.569	0.041	0.62
	95% CI	56.00–66.57	60.01–71.05	59.64–70.64	61.92–73.19	61.11–72.28	1.87–8.44		55.48–65.76	61.93–72.94	62.00–73.02	64.90–76.25	65.56–77.07	5.65–14.64				
KOOS symptoms & stiffness score	Mean	61.78	66.55	65.21	69.01	65.67	5.05	0.263	61.40	66.24	67.77	71.48	69.96	9.12	0.001	0.574	0.227	0.36
	95% CI	56.72–67.28	61.24–72.32	59.97–70.90	63.57–74.92	60.41–71.39	2.24–11.41		56.46–66.78	61.05–71.88	62.50–73.49	66.01–77.41	64.51–75.86	5.26–15.82				
KOOS daily living score	Mean	67.85	70.17	72.10	73.24	72.85	6.17	0.111	70.73	73.40	73.92	77.03	77.85	8.26	0.008	0.855	0.315	0.28
	95% CI	62.84–73.25	65.05–75.69	66.89–77.71	67.98–78.9	67.61–78.50	3.95–9.65		65.69–76.15	68.24–78.94	68.74–79.49	71.71–82.74	72.45–83.65	5.53–12.34				
KOOS sports & recreation score	Mean	35.36	37.15	40.14	40.99	43.46	6.49	0.012	38.66	44.38	49.74	49.60	52.43	12.90	<.001	0.747	0.046^d^	0.57
	95% CI	29.44–42.47	31.02–44.49	33.64–47.88	34.39–48.85	36.56–51.66	3.72–11.33		32.45–46.05	37.5–52.51	42.23–58.59	42.10–58.43	44.54–61.73	8.50–19.60				
KOOS quality of life score	Mean	37.90	40.99	39.56	42.83	45.34	7.88	<.001	44.04	44.75	44.72	48.89	50.08	5.76	0.005	0.670	0.384	-0.25
	95% CI	32.56–44.11	35.28–47.62	34.03–46.00	36.91–49.71	39.11–52.56	4.93–12.60		38.09–50.91	38.72–51.71	38.69–51.68	42.37–56.4	43.41–57.78	3.39–9.80				

Results (estimated means) are generated from linear mixed-effects models adjusted for age, sex, BMI, and CTTES positive expectancies score.

^a^ *P*-values represent within-group changes, generated from repeated measures generalised mixed-effects models adjusted for age, sex, BMI, and CTTES positive expectancies score.

^b^ *P*-values represent time × group interactions, generated from repeated measures generalised mixed-effects models adjusted for age, sex, BMI, and CTTES positive expectancies score.

^c^ *P*-values represent between-group differences in change in scores (from day 0 to 28) using linear mixed-effects models adjusted for age, sex, BMI, CTTES positive expectancies score, and corresponding baseline scores.

^d^ Under the hierarchical testing procedure, this finding is classed as non-significant.

**Figure 3:**
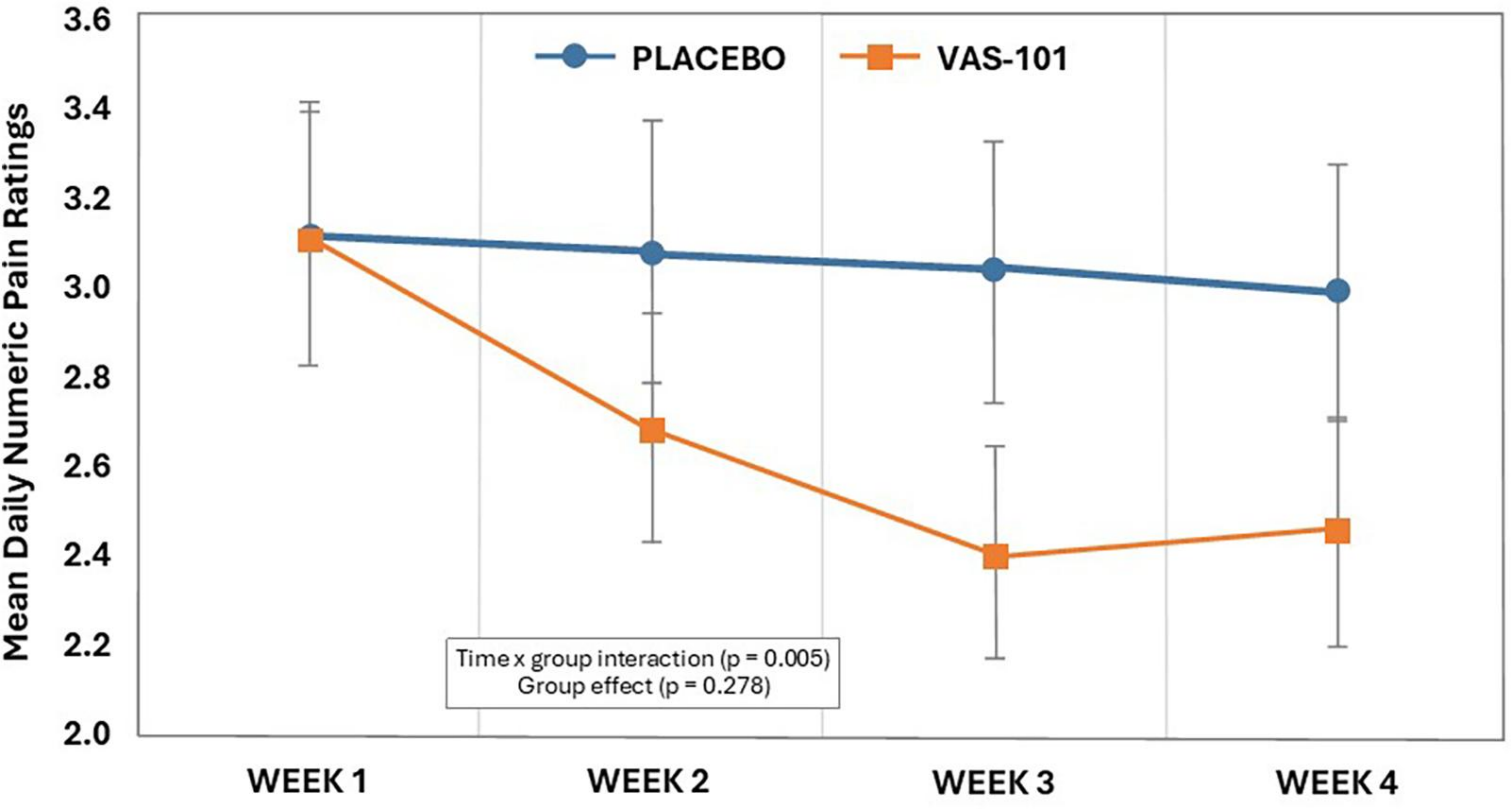
Mean weekly NPRS ratings (vertical bars represent standard errors) (estimated marginal means).

Mean daily pain ratings of the FAS are detailed in Table 3 and Figure 3. Based on the GLMM, a statistically significant interaction between time and group was observed (F_3, 225_ = 4.42, d = 0.55, *p* = 0.005), but a non-significant group effect (F_1, 225_ = 1.18, *p* = 0.278). In the VAS-101 group, the mean daily NPRS decreased from 0.65 points from week 1 to week 4 (CI: 0.27, 1.04, *p* < 0.001), and in the placebo group, there was a non-significant change of 0.14 points (CI: −0.20, 0.48, *p* = 0.983). Similar findings were obtained using the PPS (Supplementary Table S3).

**Table 3:** Change in NPRS over time (estimated marginal means) (FAS).

Group		Week 1	Week 2	Week 3	Week 4	*P*-value^a^	*P*-value^b^	*P*-value^c^
Placebo (*n* = 30)	Mean	3.12	3.08	3.04	2.99	0.983	0.005	0.278
	95% CI	2.59–3.77	2.55–3.71	2.52–3.67	2.47–3.61			
VAS-101 (*n* = 30)	Mean	3.11	2.69	2.41	2.46	<.001		
	95% CI	2.59–3.75	2.22–3.25	1.98–2.94	2.02–3.00			

Results (estimated means) are generated from generalised mixed-effects models adjusted for age, sex, BMI, and CTTES positive expectancies score.

^a^ *P*-values represent within-group changes from week 1 to week 4, generated from repeated measures generalised mixed-effects models adjusted for age, sex, BMI, and CTTES positive expectancies score.

^b^ *P*-values represent time × group interactions, generated from repeated measures generalised mixed-effects models adjusted for age, sex, BMI, and CTTES positive expectancies score.

^c^ *P*-value represents group effects, generated from repeated measures generalised mixed-effects models adjusted for age, sex, BMI, and CTTES positive expectancies score.

An examination of the percentage of participants achieving an MCID based on the KOOS pain score (change from baseline > 15.4) revealed that 13.3% (4 out of 30 participants) in the placebo group achieved an MCID, compared with 32.1% (9 out of 28 participants) in the VAS-101 group. Based on a chi-square test, this result was not statistically significant [*χ*^2^(1, *N* = 50) = 3.32, *p* = 0.086].

#### 3.2.2 KOOS subscale scores

As demonstrated in Table 2 and Figure 2, there was a statistically significant group difference in the change in the KOOS Sports and Recreation score from baseline to day 28 (β: 6.41; 95% CI: 0.12, 12.7; d = 0.57, *p* = 0.046). However, under the hierarchical testing procedure, this finding is classed as non-significant. In the VAS-101 group, the sports and recreation score increased by a mean of 12.9 points (95% CI: 8.50, 19.60), and in the placebo group, it increased by 6.50 points (95% CI: 3.72, 11.33). There were no other statistically significant group differences in changes in the other KOOS subscale scores. Similar findings were obtained using the PPS (Supplementary Table S2).

#### 3.2.3 Performance tests

As shown in Table 4 (FAS), there were no statistically significant group differences in changes across any performance-based measures. Similar findings were obtained using the PPS (Supplementary Table S4).

**Table 4:** Change in performance-based tests over time (estimated marginal means) (FAS).

Performance task		Placebo (*n* = 30)					VAS-101 (*n* = 30)					*P*-value^b^	*P*-value^c^
		Day 0	Day 14	Day 28	Change D0 to 28	*P*-value^a^	Day 0	Day 14	Day 28	Change D0 to 28	*P*-value^a^		
Chair-Stand Test (Repetitions)	Mean	14.12	14.49	15.34	1.29	0.107	13.01	13.39	14.04	0.83	0.165	0.980	0.596
	95% CI	12.58–15.85	13.08–16.05	13.67–17.20	0.07–2.51		11.61–14.58	12.11–14.80	12.53–15.74	0.39–2.06			
Paced walk test (secs)	Mean	26.74	26.26	26.59	-0.27	1.000	26.66	26.09	25.92	−1.04	0.370	0.744	0.385
	95% CI	25.35–28.21	24.77–27.85	25.10–28.15	−1.51–0.97		25.31–28.09	24.62–27.65	24.48–27.45	−2.29–0.21			
Time-up and go test (secs)	Mean	7.20	7.11	6.80	−0.48	0.062	7.43	6.89	6.67	−0.74	<.001	0.268	0.355
	95% CI	6.71–7.73	6.62–7.63	6.33–7.30	−0.88–0.08		6.93–7.96	6.43–7.40	6.21–7.16	−1.15–0.34			
Six-minute walk (metres)	Mean	503.28	512.52	519.24	16.27	0.046	520.38	530.07	538.97	21.03	0.017	0.971	0.627
	95% CI	481.82–525.69	490.76–535.25	497.19–542.27	2.52–30.03		498.86–542.83	508.16–552.92	516.66–562.24	7.28–34.79			

Results (estimated means) are generated from linear mixed-effects models adjusted for age, sex, BMI, and CTTES positive expectancies score.

^a^ *P*-values represent within-group changes from week 1 to week 4, generated from repeated measures generalised mixed-effects models adjusted for age, sex, BMI, and CTTES positive expectancies score.

^b^ *P*-values represent time × group interactions, generated from repeated measures generalised mixed-effects models adjusted for age, sex, BMI, and CTTES positive expectancies score.

^c^ *P*-values represent between-group differences in change in scores (from day 0 to 28) using linear mixed-effects models adjusted for age, sex, BMI, CTTES positive expectancies score, and corresponding baseline scores.

#### 3.2.4 Medication use

As demonstrated in Supplementary Table S5, there were no group differences in the percentage of rescue medication-free days (z = 0.646, *p* = 0.518). Over the 28 days, participants in the placebo group did not take a rescue medication for their knee pain on 78.0% of days (21.8 days), while participants in the VAS-101 group did not take a rescue medication on 83.3% of days (23.3 days). Moreover, there were no statistically significant group differences in the total number of rescue medication pills (all types) taken over the 28 days (z = −0.826, *p* = 0.409). In the placebo group, participants ingested, on average, 21.4 pills over 28 days, while participants in the VAS-101 group consumed, on average, 11.9 pills.

#### 3.2.5 PGIC

As shown in Supplementary Table S6, on day 28, there was a statistically significant group difference in PGIC ratings, with greater improvement in the VAS-101 group than in the placebo group (Z = −2.35, *p* = 0.019). In the VAS-101 group, 11 (39.3%) participants reported feeling either much or very much improved, compared with 4 (13.3%) in the placebo group.

### 3.3 Efficacy of participant blinding

To assess the effectiveness of condition concealment during the trial, participants were asked to predict their condition allocation (i.e., placebo, VAS-101, or unsure) at the end of the study. Overall group concealment was high, as 16 (57.1%) participants in the VAS-101 group and 18 (60.0%) participants in the placebo group incorrectly predicted their group allocation or were uncertain. Only one participant correctly guessed treatment allocation where the colour of the gel was cited.

### 3.4 Adverse reactions and treatment discontinuation

No participants experienced a serious adverse event, and there were no adverse events possibly or probably related to gel administration. No participant experienced any skin irritation or allergic reaction to the gels, although skin discolouration (yellowing) was reported, which resolved two to three days after treatment discontinuation. The PGATT results are detailed in Supplementary Table S7, which demonstrates that in the VAS-101 group, 26 participants (92.9%) who completed the study reported good or excellent tolerability to the gel application, and 2 participants (7.1%) reported moderate tolerability. Moreover, there were no between-group differences in changes over time in BMI, resting pulse, or blood pressure. Two individuals from the VAS-101 group discontinued the study, but this was not related to gel administration.

## 4 Discussion

The results of this randomised, double-blind, placebo-controlled study in 60 adults with knee OA showed that, compared with placebo gel, its application once every second day for 28 days was associated with self-reported improvements in knee pain. This was evidenced by a modest 9.09-point reduction (95% CI: 5.64, 14.64; 14.9% reduction) in the KOOS pain score from baseline to day 28 in the VAS-101 group, compared to a smaller 3.97-point reduction (95% CI: 1.87, 8.44, 6.5% reduction) in the placebo group. Moreover, 32.1% of participants in the VAS-101 group achieved an MCID, compared to 13.3% in the placebo group; however, this difference did not reach statistical significance (*p* = 0.086). Compared with the placebo group, participants in the VAS-101 group experienced larger reductions in daily knee pain ratings while walking over time (*p* = 0.005), although there were no statistically significant overall group differences in ratings during the 28-day intervention (*p* = 0.278). This latter non-significant finding is at least partly due to similar knee pain ratings at week 1, after which pain ratings decreased in the VAS-101 group from week 2 onwards. On day 28, 39.3% of participants in the VAS-101 group reported feeling either much or very much improved, compared with 13.3% in the placebo group (*p* = 0.019). However, despite these positive findings, there were no group differences in changes in measures of physical performance, which included the chair-stand, paced walk, timed-up-and-go, and six-minute walk tests. There were also no group differences in changes in the KOOS subscales comprising symptoms & stiffness, daily living, and quality of life.

Reductions in self-reported pain over time, as measured by two independent self-report measures (KOOS and daily VAS ratings), provide support for the pain-reducing effects of VAS-101. However, despite greater statistically significant pain reductions in the VAS-101 group compared to placebo, clinically meaningful reductions in knee pain, as indicated by an MCID, were reported by only one-third of participants (compared to 13.3% with placebo). Therefore, for most participants, clinically meaningful reductions in pain were not obtained after 28 days of application. Moreover, as there were no changes in other KOOS scores or in the performance-based assessments, the results of this study, while positive, demonstrate modest overall efficacy with only a portion of participants experiencing clinically meaningful reductions in pain. The clinical characteristics of participants who experienced greater therapeutic efficacy could not be determined and require further investigation.

An exploratory examination of rescue medication use revealed that participants who received VAS-101 took, on average, 11.9 rescue medications (of all types) during the 28-day study period, compared to 21.4 pills in the placebo group. Group differences did not reach statistical significance, and measures of rescue medication use before treatment application were not collected. Therefore, group differences in changes in rescue medication could not be comprehensively examined. Of note, an exploratory analysis revealed that the baseline CTTES negative score (assessing worry about adverse effects from treatment) was significantly correlated with rescue medication use. That is, a more negative expectation/worry about treatment was associated with fewer medication-free days (r = −0.422, *p* < 0.001) and a greater number of rescue medications taken during the 28-day intervention (r = 0.571, *p* < 0.001). This requires further investigation, including evaluation of pre-treatment use and more comprehensive assessments of medication use, and variables that are likely to influence use.

VAS-101 was well tolerated, with no treatment-related adverse reactions. The PGATT completed on day 28 revealed that in the VAS-101 group, 26 of 28 participants who completed the study reported good or excellent tolerability to the gel application, while 2 participants reported moderate tolerability. However, some participants reported concerns about skin and clothing staining, although this did not lead to treatment discontinuation in any participants. While positive, longer trials will be required to determine the long-term acceptability and adherence to the treatment.

The topical application of NSAIDs has been more extensively evaluated for the treatment of knee OA. In a meta-analysis of 14 placebo-controlled studies investigating the topical application of diclofenac (9 studies), ketoprofen (2 studies), and ibuprofen (3 studies) on self-reported pain, an overall effect size of 0.365 (95% CI 0.240, 0.490) was identified, with effect sizes ranging from 0.02 to 1.05 (30). An effect size of 0.62 on the KOOS pain score was observed in this study, suggesting at least comparable efficacy. However, direct comparisons are confounded by differences in treatment duration, where topical NSAIDs were applied daily for 1–12 weeks. Moreover, no studies, including the present study, have evaluated the duration of symptom relief after treatment cessation.

Only one study has been identified that investigated the topical application of curcumin for knee OA. In this study, a 5% curcumin ointment was applied topically to the target knee of participants aged 60 years or older with knee OA (31). However, in contrast to this current study, 1.5 mL of the ointment (vs. 0.1 mL) was applied twice daily (vs. once every second day) for 6 weeks (vs. 28 days). Moreover, changes in knee pain were assessed only using a visual analogue scale, and questions remain about blinding efficacy, as Vaseline without colouring was used as the placebo. In the current study, blinding efficacy was confirmed, as 57% of participants in the VAS-101 group and 60% in the placebo group incorrectly predicted their group allocation or were unsure. Only one participant correctly guessed the treatment allocation, citing the gel's colour. While it is difficult to directly compare these studies due to differences in gel composition, treatment duration, and treatment regimens, the use of multiple outcome measures and a more robust placebo control extends the findings from the previous study.

Although there have been no investigations comparing oral vs. transdermal delivery of curcumin for knee OA, this is the second study examining the effects of a curcumin extract, Curcugen®, on knee OA. However, in contrast to this study, where Curcugen® was applied topically at a dose of 0.1mls (8 mg) every second day for 28 days, in the previous study, it was delivered orally at a dose of 1,000 mg daily for 8 weeks (32). Comparisons between the two studies need to be undertaken cautiously due to differences in the recruited populations, such as differences in mean age (oral = 58 vs. topical = 62 years), sex distribution (oral = 50% vs. topical = 38% males), and BMI (oral = 29 vs. topical = 27 kg/m^2^). However, in both studies, statistically significant group differences were identified in the KOOS pain score, with a Cohen's D effect size of 0.39 in the oral study and a nominally larger effect size of 0.62 in this topical study. In the present study, 32.1% (9 out of 28 completed) achieved an MCID based on the KOOS pain score, compared with 33% in the oral study. However, group differences were identified in the timed-up-and-go test and the 6-minute walk test in the oral study, but none were found in the present study. No treatment-related adverse events were identified in the present study; however, mild-severity digestive complaints were reported in the oral study. While comparisons between the two studies are difficult, both forms of delivery have demonstrated some efficacy in treating OA symptoms, and investigations into differences in efficacy, tolerability, and acceptance between oral and transdermal delivery are warranted.

### 4.1 Strengths, limitations and directions for future research

Evidence of pain-relieving effects from two independent sources of self-report data and the good tolerability of VAS-101, demonstrated using a robust, randomised, double-blind, placebo-controlled study design, are strengths of this study. Moreover, confirmation of successful blinding through questioning all participants at the end of the study, and the assessment and control of participant expectations through the administration of the CTTES at baseline, are additional strengths of the study. However, several limitations of the study and recommendations for future research are provided.

This study comprised a small sample of 60 participants, so future trials utilising larger sample sizes will be important to verify the findings from the present study. In particular, several secondary and exploratory analyses were undertaken, which increases the risk of type 1 errors. Therefore, these hypothesis-generating analyses, such as the use of rescue medication and functional assessments, require further investigation. Moreover, larger sample sizes will help identify patient characteristics more likely to benefit from VAS-101 administration, such as age, sex, BMI, treatment expectancies, and OA duration and severity.

While a greater proportion of participants in the curcumin group achieved an MCID than in the placebo group (32.1% vs. 13.3%), approximately three-quarters of participants in the VAS-101 group did not achieve an MCID. Variations in application regimens will help determine if greater therapeutic efficacy and higher rates of MCID can be achieved. In this study, 0.1 mL of VAS-101 was applied every second day, removed after approximately 10–12 h, and administered for 28 days. Pharmacokinetic studies, further clinical trials, and ongoing consumer feedback will be important for determining the optimal and practical dosing regimen to achieve the greatest treatment efficacy and the highest treatment compliance. Although no participant withdrew due to difficulties around administration, higher dropout rates and treatment non-compliance are likely with extended treatment. Skin and clothing discolouration are weaknesses associated with current treatment administration, so the treatment's compliance and acceptability, particularly when used as a longer-term treatment, require further investigation. This limitation may be overcome by administering it via alternative delivery forms, such as adhesive patches, or applying the gel for shorter time periods (e.g., 60 min). In a preclinical study, peak concentrations of curcumin were detected in plasma 60 min after application (12).

It is worth noting that typical treatments and clinical investigations for OA typically last 3 months. Positive pain-relieving effects were observed after 28 days; however, a longer trial of at least 3 months will be necessary to assess changes in knee pain and other OA symptoms, as well as treatment adherence and adverse effects. Moreover, no changes in functional outcomes were identified in this study, which may be attributed to the short treatment period. Longer trials of at least 3 months will be required to determine if chronic treatment results in changes in functional outcomes. In addition, alternative performance-based assessments should be considered in future trials. Anecdotal reports from investigators suggested that participants’ efforts to complete tasks varied significantly between visits. This is evidenced by nominally inferior performance on the paced-walk test during visits 1 and 3 in almost half of participants, despite an overall average reduction in self-reported knee pain. However, it is important to note that this observation about variability in participants’ efforts is speculative, and factors such as insufficient duration, dose, or the sensitivity of the functional measures are likely to play important roles in the non-significant findings. The use of more controlled assessments, where intensity can be controlled, such as treadmill or stationary bicycle-based assessments, may be advantageous. Moreover, self-reported pain during such evaluations will be important to determine whether these tasks are associated with reduced pain.

Although rescue medication use was monitored over time, exploratory analyses were conducted. Therefore, in future trials, a more comprehensive monitoring system will help reliably identify changes in its use. This includes the role of treatment expectancies, which were significantly correlated with rescue medication use. Objective assessments will also help determine structural changes and mechanisms of action associated with topical curcumin application. These include blood/urinary markers of inflammation and oxidative stress, cartilage formation/degradation, functional magnetic resonance imaging, quantitative sensory testing, and pressure pain thresholds (33–36). Given VAS-101's pain-relieving effects, its use for other acute or chronic pain conditions, such as delayed-onset muscle soreness, acute injuries, back pain, or other OA conditions, may be useful. Finally, a further follow-up after treatment cessation will be important to determine if any treatment-related gains are maintained.

Although more research is required to better understand curcumin's mechanistic actions in the treatment of knee OA, it is postulated that its benefits when taken orally arise from its anti-inflammatory, antioxidant, protective, and anti-apoptotic effects on chondrocytes (37, 38). Mechanistic actions arising from transdermal delivery were not investigated in this study and require investigation in future trials.

## 5 Conclusions

The results of this randomised, double-blind, placebo-controlled, 28-day study demonstrated that the topical application of a novel curcumin gel (VAS-101) was associated with modest reductions in self-reported knee pain in adults with knee OA. Approximately one-third of participants who applied VAS-101 experienced clinically meaningful reductions in self-reported pain, compared with 13% who applied the placebo, although this group difference did not reach statistical significance. This study is the second to demonstrate the beneficial effects of topical curcumin application. However, the robustness of the findings is affected by the short treatment duration, the small sample size, and inconsistencies across the different outcome measures, particularly the self-report and functional measures. Further studies with larger sample sizes, variations in treatment regimens, and the use of objective outcome measures will be important to consolidate and extend the findings of the present study.

## Data Availability

The raw data supporting the conclusions of this article will be made available by the authors, without undue reservation.
