# Supplementary material for: The effect of a topical curcumin formulation (VAS-101) on knee pain in adults with knee osteoarthritis: a randomised, double-blind, placebo-controlled study
Source: Front Pain Res (Lausanne). 2026 Apr 1;7:1789088. doi: 10.3389/fpain.2026.1789088 (PMC13079332; doi:10.3389/fpain.2026.1789088)
Supplement: Supplementary file 1 [file Table1.docx]

Supplementary Material

# The effect of a topical curcumin formulation (VAS-101) on knee pain in adults with knee osteoarthritis: A randomised, double-blind, placebo-controlled study

Adrian L Lopresti^1,2*^, Benny Antony^3,4^, Stephen J Smith^1^

^1^Clinical Research Australia, Perth, Western Australia, 6023, Australia

^2^College of Science, Health, Engineering and Education, Murdoch University, Perth, Western Australia, 6150, Australia

^3^Menzies Institute for Medical Research, University of Tasmania, Hobart, Tasmania 7000, Australia.

^4^Kolling Institute, Faculty of Medicine and Health, The University of Sydney, St Leonards, New South Wales 2064, Australia

*** Correspondence:**Corresponding Author: Adrian Lopresti, Clinical Research Australia, 38 Arnisdale Road, Duncraig, Western Australia 6023, Australia adrian@clinicalresearch.com.au

## Supplementary Table 1. Hierarchical structure of endpoints

| **Rank** | **Endpoints** | **Time point** | **P-value** | **Statistically significant? *** |
| --- | --- | --- | --- | --- |
| **Primary endpoint** | | | | |
| 1 | Change in KOOS Pain Score | Day 0 to 28 | 0.041 | Y |
| **Pre-specified order of secondary endpoints** | | | | |
| 1 | VAS Daily Pain score | Weekly means | 0.005 | Y |
| 2 | PGIC ratings | Day 28 | 0.019 | Y |
| 3 | MCID, Change in KOOS Pain Score | Day 0 to 28 | 0.086 | N |
| **Secondary/ Exploratory endpoints (order not pre-specified)** | | | | |
| 1 | Change in Chair-Stand Test (Repetitions) | Day 0 to 28 | Not applicable | N |
| 2 | Change in Paced walk test (secs) | Day 0 to 28 | Not applicable | N |
| 3 | Change in Time-up and go test (secs) | Day 0 to 28 | Not applicable | N |
| 4 | Change in Six-minute walk (metres) | Day 0 to 28 | Not applicable | N |
| 5 | Change in KOOS Symptoms and Stiffness score | Day 0 to 28 | Not applicable | N |
| 6 | PGIC ratings | Day 14 | Not applicable | N |
| 7 | Total number of medication pills taken | Day 0 to 28 | Not applicable | N |
| 8 | Number of medication-free days | Day 0 to 28 | Not applicable | N |
| 9 | Change in KOOS Daily Living score | Day 0 to 28 | Not applicable | N |
| 10 | Change in KOOS Sports and Recreation score | Day 0 to 28 | Not applicable | N |
| 11 | Change in KOOS Quality of Life Scores score | Day 0 to 28 | Not applicable | N |

* Statistical significance was no longer reported after the first endpoint failed to reach statistical significance (p > 0.05)

## Supplementary Table 2. Change in KOOS Scores (estimated marginal means) (PPS)

|  | | | Placebo (n=30) | | | | | | | | | | | VAS-101 (n=28) | | | | | | | P-value^b^ | P-value^c^ | Cohen's D |
| --- | --- | --- | --- | --- | --- | --- | --- | --- | --- | --- | --- | --- | --- | --- | --- | --- | --- | --- | --- | --- | --- | --- | --- |
|  |  |  | Day 0 | | Day 7 | | Day 14 | Day 21 | | Day 28 | | Change D0 to 28 | P-value^a^ | Day 0 | Day 7 | Day 14 | Day 21 | Day 28 | Change D0 to 28 | P-value^a^ |  |  |  |
| KOOS Pain Score | Mean | 61.40 | | 66.12 | | 65.38 | | 67.40 | 66.67 | | 3.97 | | 0.008 | 61.37 | 67.26 | 68.34 | 71.64 | 72.24 | 9.09 | <.001 | 0.462 | 0.041 | 0.62 |
|  | 95% CI | 56.62-66.70 | | 60.97-71.70 | | 60.28-70.92 | | 62.18-73.06 | 61.49-72.28 | | 1.87-8.44 | |  | 56.43-66.73 | 62.00-72.96 | 63.02-74.11 | 66.13-77.61 | 66.70-78.25 | 5.64-14.64 |  |  |  |  |
| KOOS Symptoms & Stiffness Score | Mean | 61.70 | | 66.49 | | 65.15 | | 68.95 | 65.62 | | 4.10 | | 0.080 | 61.29 | 65.31 | 67.57 | 71.47 | 69.83 | 8.97 | <.001 | 0.443 | 0.159 | 0.40 |
|  | 95% CI | 56.56-67.32 | | 61.07-72.37 | | 59.81-70.97 | | 63.40-75.00 | 60.26-71.47 | | -0.74-8.94 | |  | 56.11-66.94 | 59.91-71.20 | 62.04-73.60 | 65.72-77.74 | 64.17-75.99 | 4.10-13.84 |  |  |  |  |
| KOOS Daily Living Score | Mean | 67.79 | | 70.12 | | 72.09 | | 72.97 | 72.61 | | 4.77 | | 0.027 | 71.59 | 73.02 | 74.72 | 78.09 | 78.75 | 7.19 | 0.002 | 0.782 | 0.140 | 0.41 |
|  | 95% CI | 62.77-73.20 | | 64.99-75.65 | | 66.87-77.72 | | 67.71-78.65 | 67.36-78.27 | | 3.17-7.18 | |  | 66.35-77.25 | 67.71-78.74 | 69.32-80.53 | 72.53-84.08 | 73.16-84.77 | 4.97-10.41 |  |  |  |  |
| KOOS Sports and Recreation Score | Mean | 35.61 | | 37.33 | | 40.37 | | 41.22 | 43.73 | | 6.05 | | 0.003 | 38.29 | 45.10 | 49.39 | 50.59 | 52.90 | 12.64 | <.001 | 0.618 | 0.034 | 0.60 |
|  | 95% CI | 29.62-42.82 | | 31.13-42.7 | | 33.79-48.22 | | 34.54-49.19 | 36.74-52.05 | | 3.46-10.57 | |  | 31.92-45.94 | 37.91-53.66 | 41.66-58.55 | 42.71-59.92 | 44.73-62.56 | 8.25-19.35 |  |  |  |  |
| KOOS Quality of Life Score | Mean | 37.74 | | 40.81 | | 39.39 | | 42.65 | 45.14 | | 7.06 | | <.001 | 45.07 | 45.41 | 46.11 | 50.33 | 51.37 | 5.12 | 0.001 | 0.520 | 0.520 | -0.27 |
|  | 95% CI | 32.49-43.84 | | 35.20-47.31 | | 33.94-45.70 | | 36.82-49.40 | 39.02-52.23 | | 4.52-11.03 | |  | 38.90-52.21 | 39.21-52.60 | 39.83-53.39 | 43.55-58.18 | 44.46-59.36 | 3.20-8.19 |  |  |  |  |

Results (estimated means) are generated from linear mixed-effects models adjusted for age, sex, BMI, and CTTES positive expectancies score.

^a^P-values represent within-group changes, generated from repeated measures generalised mixed-effects models adjusted for age, sex, BMI, and CTTES positive expectancies score.

^b^P-values represent time x group interactions, generated from repeated measures generalised mixed-effects models adjusted for age, sex, BMI, and CTTES positive expectancies score.

^c^P-values represent between-group differences in change in scores (from day 0 to 28) using linear mixed-effects models adjusted for age, sex, BMI, CTTES positive expectancies score, and corresponding baseline scores.

##

## Supplementary Table 3. Change in NPRS over time (estimated marginal means) (PPS)

|  | | Week 1 | Week 2 | Week 3 | Week 4 | P-value^a^ | P-value^b^ |
| --- | --- | --- | --- | --- | --- | --- | --- |
| Placebo (n=30) | Mean | 3.15 | 3.10 | 3.06 | 3.01 | 0.952 | 0.003 |
|  | SE | 0.30 | 0.30 | 0.29 | 0.29 |  |  |
| VAS-101 (n=28) | Mean | 3.18 | 2.73 | 2.44 | 2.50 | < .001 |  |
|  | SE | 0.31 | 0.28 | 0.26 | 0.26 |  |  |

Results (estimated means) are generated from generalised mixed-effects models adjusted for age, sex, BMI, and CTTES positive expectancies score.

^a^P-values represent within-group changes from week 1 to week 4, generated from repeated measures generalised mixed-effects models adjusted for age, sex, BMI, and CTTES positive expectancies score.

^b^P-value represents time x group interactions, generated from repeated measures generalised mixed-effects models adjusted for age, sex, BMI, and CTTES positive expectancies score.

##

## Supplementary Table 4. Change in Performance-Based Tests Over Time (estimated marginal means) (PPS)

|  | | | Placebo (n=28) | | | | | VAS-101 (n=28) | | | | |  | P-value^c^ |
| --- | --- | --- | --- | --- | --- | --- | --- | --- | --- | --- | --- | --- | --- | --- |
|  |  |  | Day 0 | Day 14 | Day 28 | Change  D0 to 28 | P-value^a^ | Day 0 | Day 14 | Day 28 | Change  D0 to 28 | P-value^a^ | P-value^b^ |  |
| Chair-Stand Test (Repetitions) | Mean | 14.03 | | 14.60 | 15.73 | 1.64 | 0.003 | 12.76 | 13.25 | 13.85 | 0.83 | 0.030 | 0.754 | 0.299 |
|  | SE | 0.83 | | 0.86 | 0.93 | 0.55 |  | 0.74 | 0.77 | 0.81 | 0.54 |  |  |  |
| Paced walk test (secs) | Mean | 26.74 | | 26.40 | 25.84 | -0.93 | 0.041 | 26.73 | 26.00 | 25.70 | -1.01 | 0.024 | 0.877 | 0.870 |
|  | SE | 0.77 | | 0.76 | 0.75 | 0.38 |  | 0.76 | 0.74 | 0.74 | 0.37 |  |  |  |
| Time-up and go test (secs) | Mean | 7.17 | | 7.17 | 6.62 | -0.62 | 0.002 | 7.46 | 6.90 | 6.67 | -0.74 | <.001 | 0.215 | 0.613 |
|  | SE | 0.27 | | 0.28 | 0.22 | 0.17 |  | 0.28 | 0.26 | 0.21 | 0.16 |  |  |  |
| Six-minute walk (metres) | Mean | 502.14 | | 510.67 | 521.29 | 19.46 | 0.004 | 522.82 | 532.65 | 541.32 | 20.81 | 0.006 | 0.958 | 0.886 |
|  | SE | 11.50 | | 11.66 | 11.86 | 6.62 |  | 11.66 | 11.84 | 11.99 | 6.52 |  |  |  |

Results (estimated means) are generated from linear mixed-effects models adjusted for age, sex, BMI, and CTTES positive expectancies score

^a^P-values represent within-group changes from week 1 to week 4, generated from repeated measures generalised mixed-effects models adjusted for age, sex, BMI, and CTTES positive expectancies score.

^b^P-values represent time x group interactions, generated from repeated measures generalised mixed-effects models adjusted for age, sex, BMI, and CTTES positive expectancies score.

^c^P-values represent between-group differences in change in scores (from day 0 to 28) using linear mixed-effects models adjusted for age, sex, BMI, CTTES positive expectancies score, and corresponding baseline scores.

##

## Supplementary Table 5. Intake of Rescue Medication for Knee OA during the study period

| Group | | N | Mean | SE | p-value* |
| --- | --- | --- | --- | --- | --- |
| Percentage of Rescue Medication-Free Days | Placebo | 30 | 78.02 | 6.66 | 0.518 |
|  | VAS-101 | 30 | 83.27 | 6.05 |  |
| Total Number of Rescue Medication Pills (all types) Taken over 28 days | Placebo | 30 | 21.40 | 7.40 | 0.409 |
|  | VAS-101 | 30 | 11.93 | 4.96 |  |

* p-value using an Independent-Samples Mann-Whitney U test.

## Supplementary Table 6. Frequency PGIC on Days 14 and 28

|  | | Placebo | Curcumin | P-value* |
| --- | --- | --- | --- | --- |
| PGIC Day 14 | Much Improved | 2 (6.7%) | 6 (20.0%) | 0.069 |
|  | Minimally Improved | 9 (30.0%) | 10 (33.3%) |  |
|  | No Change | 16 (53.3%) | 14 (46.7%) |  |
|  | Minimally Worse | 2 (6.7%) | 0 (0.0%) |  |
|  | Much Worse | 1 (3.3%) | 0 (0.0%) |  |
| PGIC Day 28 | Very Much Improved | 2 (6.7%) | 1 (3.6%) | 0.019 |
|  | Much Improved | 2 (6.7%) | 10 (35.7%) |  |
|  | Minimally Improved | 7 (23.3%) | 7 (25.0%) |  |
|  | No Change | 17 (56.7%) | 10 35.7%) |  |
|  | Minimally Worse | 1 (3.3%) | 0 (0.0%) |  |
|  | Much Worse | 1 (3.3%) | 0 (0.0%) |  |

* p-value using an independent samples Mann-Whitney U Test

## Supplementary Table 7. Frequency of PGATT Responses at Days 14 & 28

|  | **Placebo (n)** | **VAS-101 (n)** | **P-value*** |
| --- | --- | --- | --- |
| **Day 14** | | | |
| MODERATE. I experienced moderate discomfort/ side effects, and it had some effect on my normal activities | 0 (0.0%) | 2 (6.7%) | 0.105 |
| GOOD. I experienced minimal discomfort/ side effects, but it did not interfere with my normal activities | 0 (0.0%) | 1 (3.3%) |  |
| EXCELLENT. I experienced no discomfort or adverse effects | 30 (100.0%) | 27 (90.0%) |  |
| **Day 28** | | | |
| MODERATE. I experienced moderate discomfort/ side effects, and it had some effect on my normal activities | 0 (0.0%) | 2 (7.1%) | 0.163 |
| GOOD. I experienced minimal discomfort/ side effects, but it did not interfere with my normal activities | 1 (3.3%) | 3 (10.0%) |  |
| EXCELLENT. I experienced no discomfort or adverse effects | 29 (96.7%) | 23 (82.1%) |  |

* Chi-square test
